# The tumor microenvironment shapes gastric cancer progression by coordinating immune suppression and metabolic reprogramming

**DOI:** 10.3389/fimmu.2026.1787060

**Published:** 2026-03-11

**Authors:** Fuzhi Jiao, Zhen Wang, Jing Yuan, Fenglei Shi, Shengnan Zhang

**Affiliations:** 1 Department of Traditional Chinese Medicine, Qingdao Municipal Hospital, Qingdao, Shandong, China; 2 School of Biological and Chemical Engineering, Qingdao Technical College, Qingdao, Shandong, China

**Keywords:** extracellular matrix remodeling, gastric cancer, immune evasion, metabolic reprogramming, regulatory T cells, tumor microenvironment, tumor-associated macrophages

## Abstract

Gastric cancer (GC) remains a leading cause of cancer mortality, largely owing to metastasis driven by a highly dynamic tumor microenvironment (TME). Immunosuppressive regulatory T cells (Tregs) and tumor-associated macrophages (TAMs) orchestrate immune evasion through checkpoint signaling and polarization programs, while cancer-associated fibroblasts (CAFs) reshape stromal architecture and promote hypoxia. Concurrently, ECM remodeling—mediated by integrins, growth factors, and matrix metalloproteinases—activates oncogenic pathways such as PI3K/AKT/mTOR, MAPK/ERK, and TGF-β to drive dissemination. Metabolic reprogramming, including glycolysis-derived lactate accumulation, fatty acid and cholesterol dysregulation, and altered amino acid utilization, further constrain antitumor immunity and support angiogenesis and therapeutic resistance. This review summarizes recent advances in the bidirectional crosstalk between GC cells and key TME components, emphasizing how immune remodeling, extracellular matrix (ECM) reprogramming, and metabolic rewiring converge to sustain tumor progression, while highlighting integrative signaling networks linking immune cells, ECM, and metabolites, and providing emerging opportunities for multi-target strategies that disrupt TME-dependent metastasis.

## 1 Introduction

Gastric cancer (GC) remains one of the most prevalent malignancies worldwide and continues to impose a substantial burden on human health (1). Despite considerable advances in diagnostic modalities and therapeutic interventions, mortality from GC remains unacceptably high (2). Importantly, most GC-related deaths arise not from the primary lesion itself, but from metastatic dissemination (3). GC progression is a dynamic and multistep process shaped by reciprocal interactions between malignant cells and the tumor microenvironment (TME) (4). As a central determinant of tumor growth and metastasis, the TME engages in complex and highly coordinated signaling crosstalk with GC cells, thereby critically influencing disease progression (5, 6). The TME comprises stromal and immune cell populations, extracellular matrix (ECM), vasculature, and other non-malignant components, all of which communicate with GC cells through diverse signaling pathways to regulate key malignant phenotypes (7–9).

Although previous reviews have addressed the contribution of the TME to GC progression, a major conceptual gap remains in understanding how immune modulation, metabolic reprogramming, and ECM remodeling function as an integrated regulatory network (10, 11). This review outlines that gap by emphasizing the immune–metabolic–ECM axis as a unifying framework for interpreting the multilayered interactions that drive GC progression. In particular, this integrative perspective highlights how coordinated changes across these three dimensions promote immune evasion, therapeutic resistance, and metastatic potential. Elucidating how immune cell states, metabolic cues, and ECM dynamics converge to shape tumor behavior may refine our understanding of GC biology and identify actionable vulnerabilities.

## 2 Interaction between gastric cancer and the tumor microenvironment

### 2.1 Immunosuppressive effects of immune cells on gastric cancer

Regulatory T cells (Tregs) constitute a specialized subset of T lymphocytes with potent immunosuppressive activity. According to developmental origin and biological properties, Tregs are broadly classified into natural regulatory T cells (nTregs) and induced regulatory T cells (iTregs) (12, 13). nTregs arise in the thymus and maintain immune tolerance through transcriptional programs involving factors such as NF-κB, whereas iTregs are generated in peripheral tissues and can be induced by inhibitory cytokines, including IL-2 and TGF-β, within the tumor microenvironment, thereby facilitating immune evasion by gastric cancer cells (14). In GC, Tregs are increasingly recognized as key drivers of disease progression and as potential prognostic biomarkers. They suppress the activity of cytotoxic T lymphocytes, natural killer cells, and other effector immune populations, thereby attenuating anti-tumor immunity, promoting immune escape, and reshaping the local immune milieu into a state permissive for tumor growth and metastatic dissemination (15). Pembrolizumab and nivolumab restore anti-tumor immune activity by enhancing immune recognition and cytotoxic responses, demonstrating clinical utility in GC (16, 17).

Tumor-associated macrophages (TAMs) represent a dominant immune cell population within the TME and are deeply involved in immune regulation, angiogenesis, and extracellular matrix remodeling, thereby exerting a central role in GC progression (18). In response to distinct cytokine and inflammatory cues, TAMs adopt divergent phenotypic and functional states, classically activated M1-like macrophages with anti-tumor properties and alternatively activated M2-like macrophages with pro-tumor functions (19). In most malignancies, TAMs predominantly exhibit M2-like characteristics, whereas only a minority display M1-like features. M2-type TAM polarization is primarily induced by cytokines such as IL-4, IL-13, macrophage colony-stimulating factor (M-CSF), and TGF-β, and these cells promote tumor progression through complex autocrine and paracrine signaling networks (20, 21). Notably, polarized M2 macrophages can secrete TGF-β, promote pre-metastatic niche formation, and enhance GC metastasis through a TGF-β/JUN/CAP2 positive feedback loop that sustains CAP2 expression; CAP2 has been identified as a key mediator of the crosstalk between GC cells and TAMs (22, 23). FGF2 has also been reported to drive macrophage polarization toward the M2/TAM phenotype. Serine protease PRSS23 enhances TAM infiltration by positively regulating FGF2 secretion, thereby promoting GC progression (24, 25). Additionally, IGFBP7 regulates TAM infiltration by enhancing FGF2 secretion and activating the FGF2/FGFR1/PI3K signaling pathway (**Table 1**) (26).

**Table 1:** Roles of tumor microenvironment in gastric cancer progression.

TME component	Cell subtype	Role	Signal	Treatment
Tumor cells	Gastric cancer cells	Proliferation, invasion, metastasis, immune escape	PI3K/AKT/mTOR, MAPK/ERK, Wnt/β-catenin, TGF-β-related EMT	Targeted inhibition of oncogenic pathways; combination strategies
Regulatory T cells (Tregs)	nTregs, iTregs	Immunosuppression and immune evasion	IL-2, TGF-β, NF-κB, PD-1/PD-L1 axis	PD-1 blockade (pembrolizumab, nivolumab)
Tumor-associated macrophages (TAMs)	M1-like, M2-like TAMs	Immune regulation, angiogenesis support, ECM remodeling, metastasis promotion	IL-4, IL-13, M-CSF, TGF-β; TGF-β/JUN/CAP2 loop; FGF2/FGFR1/PI3K/Akt; PRSS23, IGFBP7; CLEC4E	TAM-targeting strategies
Cancer-associated fibroblasts (CAFs)	CAFs	Hypoxia induction, ECM stiffening/degradation, promotion of proliferation, migration, invasion, angiogenesis	Collagen/fibronectin secretion; ECM remodeling; interaction with TAMs in collagen organization	Targeting CAF-related remodeling (general)
Extracellular matrix (ECM)	Collagen and ECM reservoir	Structural support, regulation of migration/invasion, impact on immune infiltration	Integrins (α5, β1, α5β1); MMPs; FBN1 K672 succinylation affecting MMP2-mediated degradation	Integrin α5 targeting; monoclonal antibodies against the FBN1 succinylation site
Metabolic components	Glycolysis/lactate, lipid/cholesterol, amino acids	Immune suppression, tumor-associated macrophages polarization, angiogenesis, therapy resistance	GLUT3, LDHA, HK2, PKM2, GLUT1, MCT1, LDHB, HIF-1α, PD-1; CD36,	MCT inhibition combined with immune checkpoint blockade; metabolic targeting combined with ICB
Soluble factors	Cytokines and growth factors	Immune modulation, EMT, angiogenesis, pathway activation	EGF, EGFR; TGF-β; VEGF; IL-8; ROS/NF-κB; ROS/MAPK (ERK1/2, p38)/AP-1	EGFR as a therapeutic target; TGF-β signaling inhibition to increase treatment sensitivity

### 2.2 Interaction between gastric cancer and extracellular matrix

#### 2.2.1 Influence of extracellular matrix components on gastric cancer

The ECM is a dynamic macromolecular network composed primarily of cell-secreted proteins and polysaccharides. Beyond serving as a structural scaffold, the ECM mediates bidirectional communication between cells and their surrounding microenvironment, thereby profoundly influencing core biological processes, including proliferation, differentiation, migration, and apoptosis (27, 28). As a major component of the TME, ECM dysregulation is now recognized as a defining feature of malignant transformation and progression (29). Collagen, the most abundant protein in the ECM, forms the structural framework of the extracellular matrix and is associated with tumor growth, metastasis, and the maintenance of the tumor microenvironment. In gastric cancer, collagen degradation and reorganization may facilitate tumor cell migration and invasion (30, 31). Importantly, remodeling of ECM composition and architecture can substantially influence immune cell infiltration and function, thereby contributing to tumor immune escape (10, 32). During collagen fibrillogenesis, TAMs exhibit striking functional plasticity. These cells frequently interact with cancer-associated fibroblasts (CAFs) and actively participate in ECM assembly by promoting fibrillar collagen deposition, cross-linking, and spatial organization (33, 34). Thus, macrophage–CAF crosstalk extends beyond immune regulation to directly shape tumor architecture, with important consequences for GC initiation and progression (35, 36). CAFs, which represent a major stromal component of the TME, secrete diverse ECM constituents, including collagen and fibronectin, thereby altering ECM composition and structure and exerting broad tumor-supportive functions in cancer (37). These observations underscore the close association between ECM remodeling and the biological behavior of GC cells. A deeper understanding of how specific ECM components regulate GC cell migration and invasion may provide a foundation for the development of therapeutic strategies targeting ECM abnormalities in GC.

#### 2.2.2 Signal interaction between the extracellular matrix and gastric cancer cell

The bidirectional signaling crosstalk between the ECM and GC cells involves multiple molecular classes, including matrix metalloproteinases, growth factors, and integrins (38). In parallel, intracellular signaling cascades such as the Wnt signaling pathway, PI3K/AKT pathway, and MAPK pathway play pivotal roles in transducing ECM-derived cues in GC cells (39). Integrins are heterodimeric adhesion receptors composed of α and β subunits that mediate cell–ECM adhesion and, in turn, regulate tumor proliferation, invasion, and migration (40). Among them, integrin α5β1, formed by the α5 and β1 subunits, has emerged as a key mediator of invasion and metastasis across multiple tumor types (41). Studies have shown that treatment with monoclonal antibodies or genetic ablation of integrin α5 inhibits the *in vitro* interaction between diffuse-type GC cells and CAFs, suppresses peritoneal dissemination in murine models, and correlates with patient survival (42). These findings suggest that integrin α5 may serve as a prognostic biomarker for evaluating disease progression and treatment response. In addition, the interaction between tubulointerstitial nephritis antigen-like 1 and integrin β1 accelerates the progression of diffuse-type gastric cancer (43). Growth factors such as EGF and TGF-β regulate the growth, differentiation, and migration of GC cells, playing a central role in tumor progression (44).

EGFR promotes the proliferation and development of GC, and its overexpression is associated with poor prognosis in GC, making it an important therapeutic target (45). VEGF mediates tumor angiogenesis, promoting tumor occurrence and development. Notably, high CD47 expression has been observed in GC tissues, and anti-CD47 immunotherapy has been reported to preserve the tumor angiogenic system (46). VEGF and CD47 expression levels are positively correlated in GC. Simultaneous targeting of CD47 and VEGF using SIRPα-VEGFR1, or the combination of two single-target agents, can effectively inhibit tumor growth, reduce postoperative recurrence, and significantly prolong patient survival (46). TGF-β is another key signaling node in GC progression which can promote the EMT process and confer stem cell characteristics to tumor cells, increasing treatment resistance (47). Inhibition of TGF-β signal transduction has been shown to reverse mesenchymal/stem-like phenotypes in GC cell lines and mouse xenograft models and to enhance treatment sensitivity, indicating that TGF-β is a promising therapeutic target and may provide a mechanistic basis for overcoming refractory GC (48, 49).

During cancer progression, MMPs contribute to ECM degradation, thereby dismantling structural barriers and facilitating GC cell penetration of the basement membrane, invasion of adjacent tissues, and metastatic spread (50, 51). Consistently, aberrant ECM expression and degradation are closely linked to GC initiation and clinical outcome. In 3D matrix culture systems and animal models, succinylation of FBN1 at K672 was shown to enhance FBN1 stability by impairing its interaction with MMP2, thereby reducing FBN1 degradation and promoting its accumulation in tumor tissue (52) Accumulated FBN1, in turn, promotes tumor invasion and metastasis by altering ECM structure and function, and this process is closely associated with poor prognosis in GC (53). Accordingly, monoclonal antibodies targeting the FBN1 succinylation site may represent a potentially effective therapeutic strategy by interfering with FBN1 succinylation and suppressing tumor progression.

## 3 Metabolic reprogramming of gastric cancer cells induced by immune microenvironment

### 3.1 Glucose metabolism regulates T cell activation and macrophage polarization

Metabolic reprogramming is intimately linked to the initiation and progression of gastric cancer. GC cells can promote proliferation, invasion and metastasis by reshaping metabolic homeostasis within the TME—such as glucose, amino acids, and lipids (54). In GC, glucose metabolism provides energy and serves as metabolic intermediates required for macronutrient biosynthesis, lipid metabolism helps maintain cellular homeostasis, and amino acid metabolism is involved in regulating cellular signal transduction (55, 56). This metabolic reprogramming not only affects the energy demands and biosynthesis of cancer cells themselves but also further promotes tumor immune evasion and progression by altering the function of immune cells and the activity of immune molecules in the TME (11, 57). Alterations in glycolysis lead to nutrient and energy imbalances between GC cells and immune cells in the TME, enabling tumor cells to undergo processes such as immune evasion. Therefore, the role of glycolysis in the TME cannot be underestimated. GLUT3 activates the STAT3 pathway and upregulate the expression of glycolysis-related genes, including PKM2, HK2, LDHA, and SLC2A1, thereby attenuating tumor-cell killing, reducing the activity of TILs and CD8^+^ T cells, and ultimately promoting tumor immunosuppression and GC cell proliferation (58). GLUT1 inactivation may induce metabolic reprogramming characterized by enhanced oxidative phosphorylation (OXPHOS) and excessive ROS generation, culminating in TNF-α-mediated cell death, a process associated with the occurrence and progression of gastric cancer (59, 60). The PI3K/Akt/mTOR pathway, a classic signaling pathway associated with tumor cells, is a central regulator responsible for glycolysis (61). Blocking immune checkpoints may inhibit tumor cell glycolysis, thereby enhancing the immune effects of immune cells (62).

**Figure 1:**
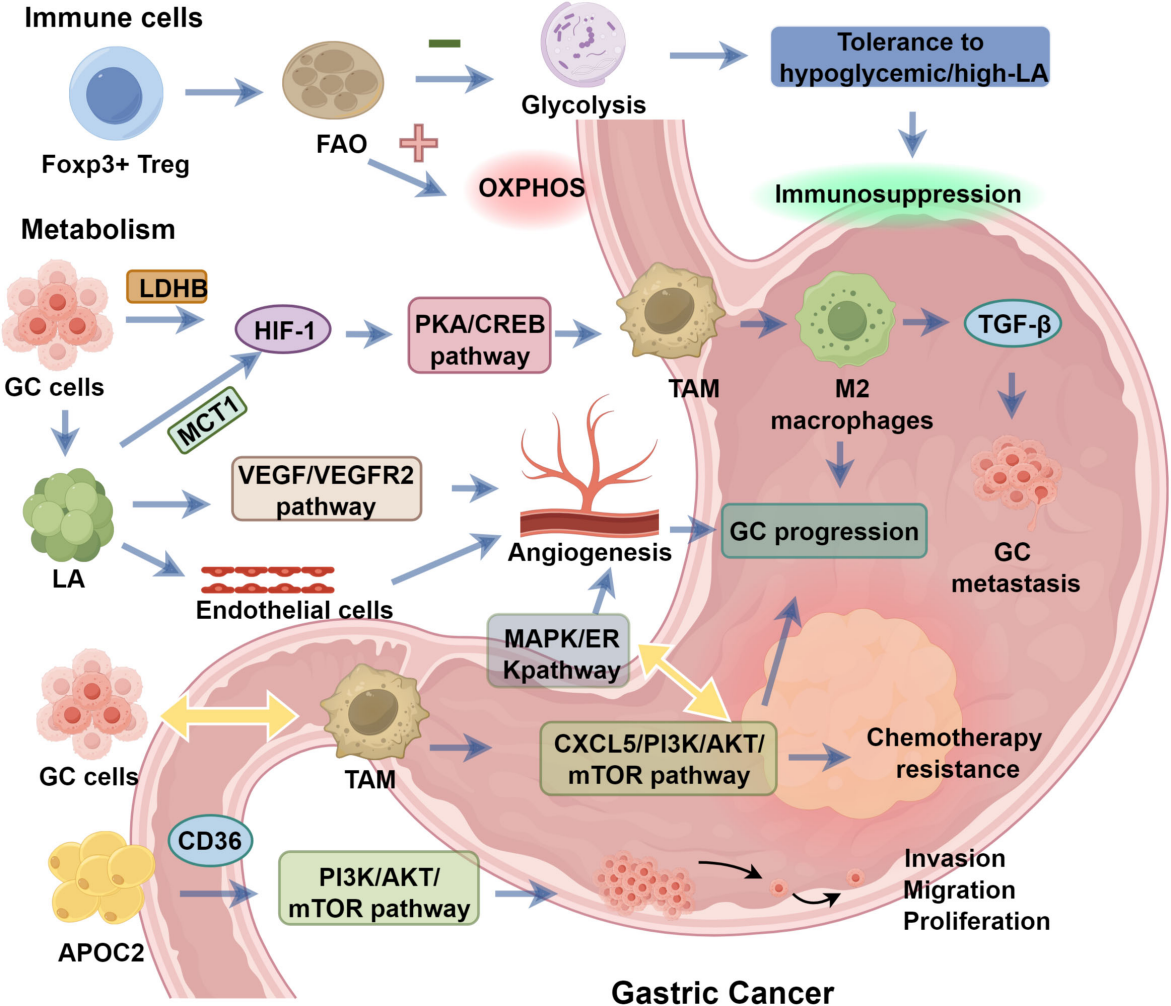
Tumor microenvironment shapes gastric cancer progression.

Lactate (LA) primarily promotes the initiation and progression of gastric cancer primarily through two interrelated mechanisms. First, high concentrations of LA inhibit the anti-tumor effects of immune cells in the TME, facilitating immune evasion of GC cells (63, 64). Tumor-infiltrating Treg cells suppress glycolysis and initiate OXPHOS metabolic reprogramming by increasing fatty acid oxidation (FAO) levels in the presence of Foxp3, thereby inducing tolerance to the hypoglycemic and high-LA characteristics of the TME, contributing to the immunosuppression in GC (65). Second, LA stabilizes HIF-1 in the hypoxic TME, promoting the polarization of TAMs toward an M2-like phenotype, which is involved in the progression of gastric cancer (66, 67). GC cells uptake LA via MCT1, and LA metabolism drives LDHB dependent HIF-1α accumulation, which in turn activates the PKA/CREB pathway to induce M2-like TAM polarization. In parallel, GC cells activate the VEGF/VEGFR2 axis or directly stimulate endothelial cells, thereby promoting tumor angiogenesis and accelerating GC growth (60, 68). Targeting MCT1 and MCT4 can enhance immune checkpoint blockade (ICB) therapy. The combination of MCT inhibitors with ICB increases TME pH and promotes immune-cell infiltration, thereby improving antitumor efficacy. Therefore, the concentration of LA in the TME is likely a key factor in tumor drug resistance, and in-depth investigation into how LA affects immune cells is crucial for optimizing immunotherapy (69, 70). In sum, highly glycolytic GC cells reshape the TME through lactate accumulation and hypoxia, reducing both the abundance and activity of immune cells, promoting immune escape, and increasing drug tolerance (**Figure 1**).

### 3.2 Lipid metabolism regulates immune cell function

Lipids play a critical role in biological membrane synthesis, cell growth, proliferation, and migration. In GC, metabolic reprogramming drives aberrant lipid metabolism, thereby disrupting cellular homeostasis and reshaping immune-cell function, with the most prominent alterations involving fatty acid (FA) transport and cholesterol homeostasis (71, 72). These metabolic derangements are tightly linked to the malignant progression of GC. At present, key FA metabolic enzymes in GC include fatty acid translocase CD36 and CPT1A, which remodel the FA metabolic network to modulate the GC tumor microenvironment (73, 74). CD36 overexpression, acting in concert with APOC2, induces epithelial–mesenchymal transition (EMT), activates the PI3K–AKT–mTOR axis to promote FA uptake in GC cells, ultimately leading to immune evasion and peritoneal metastasis of GC cells (75). Cholesterol metabolism is likewise a major determinant of immune dysfunction in GC. For example, overexpression of HMGCR in TILs leads to cholesterol accumulation in CD8^+^ T cells, which activates the endoplasmic reticulum stress sensor XBP1 and induces the production of TGF-β, VEGF, and kynurenine (Kyn). This metabolic–stress program drives the upregulation of immune checkpoints, including PD-1, accelerates CD8^+^ T-cell exhaustion and promotes immune escape in GC (76). Therefore, targeting cholesterol metabolism may influence cellular immune function. Utilizing drugs for targeted therapy to alleviate GC immunosuppression could become an emerging strategy for GC treatment (77).

### 3.3 Amino acid metabolism

Amino acids are indispensable nutrients and central substrates for protein biosynthesis. Studies have shown that arginine (Arg), tryptophan (Trp), and glutamine (Gln), their derivatives are highly expressed in GC (78, 79). GC cells reprogram the metabolism of various amino acids to support their malignant behaviors. Gln serves as a hub in multiple metabolic pathways and lays the foundation for GC malignancy by modulating cellular immune responses (80, 81). Trp metabolism contributes to immunosuppression in GC through diverse cytokine networks and signaling pathways, whereas Arg-derived metabolites perturb immune-cell states within the TME (82). Distinct amino acids converge on the PI3K/Akt/mTOR signaling axis to regulate GC initiation and progression. Gln metabolism, for instance, participates in regulating GC cell proliferation, growth, invasion, and metastasis by activating the PI3K/Akt/mTOR pathway, indicating that Gln regulation of this pathway may have a beneficial effect in suppressing gastric carcinogenesis (81, 83). In parallel, Arg metabolism can influence the development of GC by participating in the regulation of the mTOR signaling pathway (84). Targeting amino acid metabolism and related signaling pathways may represent alternative immunotherapeutic strategies for GC in the future.

## 4 Interactions among immune cells, ECM, and metabolites

The interaction between immune cells, ECM, and metabolites in the tumor microenvironment affects the activity of signaling pathways, further influencing behaviors such as proliferation, survival, and invasion of GC (85, 86). TME-related signaling pathways such as PI3K/Akt/mTOR, MAPK/ERK, and Wnt/β-catenin are closely associated with the occurrence and development of GC (87, 88). Specifically, the PI3K/Akt/mTOR signaling pathway is widely activated in GC cells and is related to apoptosis, autophagy, and survival of GC cells, promoting the proliferation, survival, and invasive ability of tumor cells (89). The interaction between TAMs and GC cells promotes chemotherapy resistance in GC through the CXCL5/PI3K/AKT/mTOR pathway. Therefore, targeting TAMs and blocking intercellular crosstalk between TAMs and GC cells may be a prospective therapeutic strategy for GC patients (90). APOC2 is a key activator of lipoprotein lipase triglyceride metabolism and participates in lipid metabolism in the TME. APOC2 promotes GC cell invasion, migration, and proliferation through CD36-mediated PI3K/Akt/mTOR signaling activation (75, 91). The MAPK/ERK signaling pathway is involved in regulating cell proliferation and angiogenesis, which is crucial for tumor growth and metastasis (92).

IL-8 secretion, mediated through the ROS/NF-κB and ROS/MAPK (ERK1/2, p38)/AP-1 axes, promotes endothelial-cell proliferation and angiogenesis within the GC tumor microenvironment (87, 88, 93). Extensive crosstalk between the PI3K/Akt/mTOR and MAPK/ERK pathways further governs GC initiation and progression (94). Notably, inhibition of PI3K/Akt/mTOR signaling can trigger compensatory activation of MAPK/ERK signaling, and vice versa, underscoring the adaptive plasticity of oncogenic signaling networks in GC. In parallel, Wnt/β-catenin signaling is closely linked to the self-renewal and differentiation capacity of gastric cancer stem cells, and may therefore contribute to tumor recurrence and therapeutic resistance (95, 96). Additional pathways, including HIF-1α signaling, are activated under hypoxic conditions and promote angiogenesis as well as metabolic adaptation, thereby supporting tumor persistence in a hostile microenvironment (97). Beyond these oncogenic and metabolic circuits, immune checkpoint pathways—particularly PD-1/PD-L1 and CTLA-4 signaling—are central mediators of tumor immune evasion and represent major targets of contemporary cancer immunotherapy (98–101). By intervening in these signaling pathways, the tumor microenvironment can be altered, tumor growth inhibited, and treatment efficacy improved.

## 5 Conclusion

Gastric cancer progression is not solely dictated by intrinsic genetic alterations, but by a highly coordinated tumor microenvironment that integrates immune suppression, extracellular matrix remodeling, and metabolic reprogramming into a unified regulatory network. Regulatory T cells and tumor-associated macrophages establish an immunosuppressive niche through checkpoint signaling and polarization programs, while cancer-associated fibroblasts and matrix remodeling reshape tissue architecture and facilitate invasion. Concurrently, glycolytic flux, lactate accumulation, lipid dysregulation, and amino acid rewiring converge on central signaling axes—including PI3K/Akt/mTOR, MAPK/ERK, Wnt/β-catenin, and HIF-1α—to reinforce immune escape, angiogenesis, and therapeutic resistance. These interconnected processes highlight the immune–metabolic–ECM axis as a core driver of GC metastasis and disease persistence.

Future therapeutic strategies should therefore move beyond single-pathway inhibition and instead target the dynamic crosstalk within the tumor microenvironment. Rational combination regimens integrating immune checkpoint blockade with metabolic modulators, ECM-targeted interventions, and signaling inhibitors may more effectively reprogram the TME and restore antitumor immunity. A deeper mechanistic understanding of how immune cells, stromal components, and metabolic cues co-evolve during GC progression will be essential for identifying actionable vulnerabilities. Ultimately, precision strategies that disrupt TME-dependent networks hold promise for overcoming immune resistance, limiting metastasis, and improving clinical outcomes in gastric cancer.
